# Development of AWaRe-Based Quality Indicators to Assess the Appropriateness of Antibiotic Prescribing in Primary Healthcare in South Africa

**DOI:** 10.3390/antibiotics15020196

**Published:** 2026-02-10

**Authors:** Audrey K. Chigome, Johanna C. Meyer, Adrian Brink, Sabiha Essack, Elmien Bronkhorst, Halima Dawood, Yasmina Johnson, Renier Coetzee, Chuma Maphathwana, Moloko Phaho, Phillip Malebaco, Nonhlanhla Nhlapo, Filip Djukic, Annie Heath, Aislinn Cook, Gauri Kumar, Stephen M. Campbell, Brian Godman, Marc Mendelson

**Affiliations:** 1 Department of Public Health Pharmacy and Management, School of Pharmacy, Sefako Makgatho Health Sciences University, Ga-Rankuwa, Pretoria 0208, South Africa; hannelie.meyer@smu.ac.za (J.C.M.); stephen.campbell@smu.ac.za (S.M.C.); 2 South African Vaccination and Immunisation Centre, Sefako Makgatho Health Sciences University, Ga-Rankuwa, Pretoria 0208, South Africa; 3 Division of Medical Microbiology, Faculty of Health Sciences, University of Cape Town, Cape Town 7700, South Africa; 4 National Health Laboratory Service, Groote Schuur Hospital, Cape Town 7925, South Africa; 5 Institute of Infectious Disease and Molecular Medicine, Faculty of Health Sciences, University of Cape Town, Cape Town 7700, South Africa; 6 Antimicrobial Research Unit, School of Health Sciences, University of KwaZulu-Natal, Durban 3629, South Africa; essacks@ukzn.ac.za; 7 Department of Clinical Pharmacy, School of Pharmacy, Sefako Makgatho Health Sciences University, Ga-Rankuwa, Pretoria 0208, South Africa; elmien.bronkhorst@smu.ac.za; 8 Infectious Diseases Unit, Department of Internal Medicine, Grey’s Hospital, Pietermaritzburg 3201, South Africa; dawoodh@ukzn.ac.za; 9 Western Cape Department of Health and Wellness, Cape Town 8001, South Africa; yasmina.johnson@westerncape.gov.za; 10 School of Pharmacy, University of the Western Cape, Cape Town 7535, South Africa; 11 School of Public Health, University of the Western Cape, Cape Town 7535, South Africa; recoetzee@uwc.ac.za; 12 National Department of Health, Pretoria 0187, South Africa; vuyomaphathwana@gmail.com; 13 Bodibe New Clinic, Litchenburg 2741, South Africa; molokophaho@gmail.com; 14 Mangaung Metro District Health Services, Bloemfontein 9300, South Africa; malebacobp@fshealth.gov.za; 15 Leratswana Clinic, Arlington 9602, South Africa; nnhlapo02@gmail.com; 16 Antibiotic Policy Group, Institute for Infection and Immunity, City St. George’s, University of London, London SW17 0RE, UK; fdjukic@citystgeorges.ac.uk (F.D.); aheath@citystgeorges.ac.uk (A.H.); aicook@citystgeorges.ac.uk (A.C.);; 17 Nuffield Department of Primary Care Health Sciences, University of Oxford, Oxford OX2 6GG, UK; 18 School of Health Sciences, University of Manchester, Manchester M13 9PL, UK; 19 Division of Infectious Diseases and HIV Medicine, Department of Medicine, Groote Schuur Hospital, University of Cape Town, Cape Town 7700, South Africa; marc.mendelson@uct.ac.za

**Keywords:** antimicrobial resistance, quality indicators, RAND/UCLA appropriateness method, primary healthcare, antibiotic prescribing, infection, antibiotic stewardship, WHO AWaRe system, South Africa

## Abstract

**Background/Objectives**: The overuse and misuse of antibiotics contribute to antimicrobial resistance (AMR) globally. The appropriateness of antibiotic prescribing at the primary healthcare (PHC) level must be urgently addressed to reduce high levels of inappropriate antibiotic prescribing and associated AMR. This study aimed to develop quality indicators, based on the World Health Organization (WHO)’s Access, Watch, Reserve (AWaRe) guidance, to assess the appropriateness and quality regarding antibiotic prescribing in public PHC settings in South Africa. **Methods**: Potential indicators were identified from indicators developed by City St George’s, University of London (SGUL); a review of AWaRe-based indicators; and the results from point prevalence surveys at PHC clinics in South Africa. The indicators were developed using the RAND/UCLA Appropriateness Method. In Round 1, 12 experts individually rated 78 indicators for clarity and appropriateness. In Round 2, 10 experts rated 89 indicators for appropriateness and feasibility during an interactive online meeting. **Results**: The final set had 61/89 indicators (68.5%) that were rated both appropriate and feasible with agreement. Dental infections (9/9; 100%) alongside skin and soft tissue infections (11/13; 84.6%) had the highest percentage of indicators that were rated appropriate and feasible with agreement. Lower urinary tract infections (6/11; 54.5%) and general (4/8; 50%) categories had the lowest percentage of indicators rated appropriate and feasible with agreement. **Conclusions**: The process proved valuable in developing potential indicators for use in future antimicrobial stewardship programmes to improve antibiotic prescribing in public sector PHC facilities in South Africa and beyond.

## 1. Introduction

Antibiotics play a significant role in the prevention and treatment of infections, saving millions of lives globally [1,2]. However, effective prevention and treatment of a range of infections is threatened by increasing rates of antimicrobial resistance (AMR) across countries, exacerbated by the overuse and misuse of antibiotics [3,4,5,6,7,8,9]. As a result, bacterial infections have become the second leading cause of death globally [7]. Estimates suggest there have been up to 5 million deaths globally each year associated with bacterial resistance, and rising with increasing inappropriate antibiotic use [10,11,12].

The increase in AMR is higher among low- and middle-income countries (LMICs), including African countries, compared with high-income countries [11,13,14,15]. Similarly, the recent World Health Organization (WHO)’s global antibiotic resistance surveillance report shows that resistance to essential antibiotics is widespread, although unevenly distributed [7]. As a result, increasingly compromising the effectiveness of first line antibiotics for common infections [16]. Increasing resistance rates are enhanced by concerns with AMR surveillance and diagnostic capacity among LMICs, routine access to effective treatments, alongside the overuse of antibiotics including those from the WHO Watch and Reserve lists [17,18,19,20,21,22]. As a result, in sub-Saharan Africa alone, it is projected that there could be 4.1 million AMR-related deaths each year by 2050 unless urgent actions are undertaken [13].

To address rising AMR rates, and the implications, many countries have developed their own National Action Plans (NAPs) based on the WHO Global Action Plan on AMR [23,24]. However, among LMICs there are gaps in their implementation due to several challenges, which include resource constraints and poor implementation strategies [25,26,27,28]. Whist the authorities in South Africa previously made progress with implementing its NAP compared to other African countries, there is now an urgent need to update and reinstate the South African Antimicrobial Resistance National Strategy Framework given issues with the current lack of activities and resources, as well as concerns with rising AMR rates in the country and their implications [28,29,30,31]. Other key global activities to reduce AMR include the development and instigation of the WHO AWaRe category for antibiotics, which encourages increased use of Access antibiotics where pertinent with their lower resistance potential [32,33].

As mentioned, a key concern among LMICs adding to AMR is the increasing use of antibiotics from the Watch and Reserve categories [20,21,22,34,35], which is also seen in South Africa. Overall, there was a 50% increase from 17.9 Defined daily doses per 1000/population per day in antibiotic use between 2018 and 2022 [36], and in 2022, Watch antibiotics accounted for 52% of total consumption in the public sector compared to 48% for Access antibiotics [36]. This has fueled concerns with rising AMR rates in South Africa, and the impact on mortality, leading to recent calls to the Ministry of Health in South Africa to re-engage with the National Health Plan on AMR to reduce rising AMR rates [29,30]. The need to reverse the growing use of Watch and Reserve antibiotics in the country, and their subsequent impact on AMR, has become of increasing importance following the United Nations General Assembly’s (UN-GA) political declaration of the high-level meeting on AMR in 2024 [37]. The target is that at least 70% of overall human antibiotic use should be Access antibiotics through investing in and strengthening antimicrobial stewardship programmes (ASPs) [37].

A key sector for addressing high rates of inappropriate use among LMICs is primary care, where antibiotics can account for up to 90% or more of antibiotic use in humans [38,39]. This is important as it is currently estimated that around half of all antibiotic use is seen as inappropriate [1]. Alongside this, a systematic review found no significant decline in the prevalence of inappropriate antibiotic prescribing in primary care across countries in recent years, including LMICs, despite AMS efforts [40]. There is a similar situation in South Africa where several studies have found continued high levels of inappropriate antibiotic use across both private and public primary healthcare (PHC) centers, exacerbated by variable compliance with treatment guidelines (Supplementary Table S1) [41,42,43,44,45,46,47,48]. One of the WHO’s eight priority AMR-related interventions for strengthening PHC-oriented health systems to combat AMR globally is ensuring that antimicrobials are used appropriately and according to updated, evidence-based treatment guidelines [49]. The recent launch of the WHO AWaRe book providing prescribing guidance on a range of infectious diseases commonly seen in primary care, including non-antibiotic choices, is seen as an important development, given concerns with the potential robustness of a number of national antibiotic guidelines among LMICs [39,50]. The guidance in the WHO AWaRe book is not intended to replace existing local and national evidence-based antibiotic prescribing guidelines and clinical judgment; however, to provide guidance where currently none is available or where there are concerns with the robustness of current guidelines especially among LMICs [39,50,51]. However, antimicrobial stewardship (AMS) initiatives, alongside measuring the quality and appropriateness of antibiotic prescribing in primary care, especially among LMICs, must be prioritized going forward to reduce AMR [52].

Appropriate and feasible evidence-based quality indicators are important for sustainable improvements in antibiotic stewardship in PHCs [53,54]. Quality indicators are increasingly seen as important tools in AMS activities across sectors as they help to measure the appropriateness of antibiotic prescribing and dispensing in the treatment of infections [55,56,57,58,59]. Assessing antibiotic prescribing quality helps to foster a stewardship culture among prescribers, supports adherence to treatment guidelines, reduces variation in clinical practice, improves patient safety and clinical outcomes as well as facilitating the implementation of feedback driven quality improvement programmes [46,60,61,62].

Several indicators have been used across Africa and across the different sectors, including both hospital and primary care, as well as both private and public sectors, to improve antibiotic use in primary care [45,46,54,63]. In South Africa, examples of indicators that have been used to assess prescribing quality in PHC include the percentage (%) of prescriptions adherent to current guidelines, % of monthly antibiotics used (defined daily doses per 100 prescriptions dispensed), % of antibiotics prescribed/procured broken down by WHO AWaRe categories, and the % of patients prescribed an appropriate antibiotic dose and duration for their diagnosed infectious disease [44,45,46,63,64,65,66].

A recent international review found that a high number of quality indicators for appropriate antibiotic use have been developed; however, very few are currently based on the AWaRe system, identifying only 8 (1%) indicators directly related to the WHO AWaRe system, although 445 (57.6%) indicators reflect the guidance provided in the WHO AWaRe book [54]. This is a concern with the growing use of the WHO AWaRe classification among LMICs as a starting point for antimicrobial stewardship (AMS) activities [67].

Previous studies have used the Research and Development/University of California Los Angeles (RAND/UCLA) Appropriateness Method (RAM), which was also used in this study to develop quality indicators for assessing the appropriateness of antibiotic prescribing and use [68,69,70,71,72,73,74]. Currently though, no set of appropriate and feasible quality indicators, based on the WHO AWaRe prescribing guidance, has been specifically developed to measure the appropriateness of antibiotic prescribing in primary care in South Africa. Consequently, this study focused on the development and application of feasible and actionable quality indicators to assess the appropriateness of antibiotic prescribing in public PHC settings in South Africa. The aim of developing these indicators is to ensure that antibiotics are prescribed and used rationally and appropriately in primary care. This is important with the development of AWaRe antibiotic quality indicators for use across sectors, including primary care [66,67,68,69,70,71,72,73,74,75,76], and subsequently assessing the feasibility of their implementation [52].

## 2. Results

### 2.1. The Rating Process

For the first round, 12 panel experts individually rated 78 indicators for clarity and appropriateness. Each of the 78 statements had a median rating of at least 7, with agreement for clarity. This means that all statements were considered understandable and unambiguous. In the second round, 10 of the 12 experts (83.3%) attended the virtual consensus meeting and rated 89 indicators for appropriateness and feasibility.

Results for the second round of the RAM are presented and discussed in this paper.

### 2.2. Median Scores and Level of Consensus for Round 2

Table 1 presents a summary of the median scores and level of consensus for the 89 indicators rated in the second round, organized according to the infection categories. Overall, there was agreement for 77 indicators (86.5%) for appropriateness ratings and 75 indicators (84.3%) for feasibility ratings. Indicators in the sexually transmitted infection (STI) (12/12; 100%), dental infection (9/9; 100%) and general (8/8; 100%) categories had the highest level of agreement for appropriateness, while indicators in the bacterial eye infection category had the lowest level of agreement (*n* = 5; 62.5%). Indicators in the skin and soft tissue infection (SSTI) (13/13; 100%) and dental infection (9/9; 100%) categories had the highest levels of agreement for feasibility, while indicators in the diarrhea and enteric fever (6/9; 66.7%) and general (4/8; 50%) categories had the lowest level of agreement. Disagreement within a panel using this method is where ≥33% of panelists rated between 1–3, and ≥33% rated between 7–9, respectively, for an indicator. There was no disagreement for any indicator on either scale of appropriateness or feasibility. No indicators had a median score of 1–3 for either feasibility or appropriateness.

### 2.3. Round 2 Outcomes of Indicators Rated for Appropriateness and Feasibility

The rating outcomes for the 89 indicators rated in the second round are presented in Table 2. In total, 66 statements (74.2%) were rated appropriate and feasible with agreement. There were 11 indicators rated appropriate but not feasible (12.4%), 9 indicators were rated feasible but not appropriate (10.1%) while 3 indicators (3.4%) were rated both inappropriate and not feasible.

### 2.4. Final Set of Quality Indicators Rated Appropriate and Feasible in the Second Round

The final set of indicators rated both appropriate and feasible with agreement (*n* = 61; 68.5%) from the 89 indicators rated in the second round is presented in Table 3. In cases where both the original and rephrased statements were rated appropriate and feasible with agreement, the original statements (*n* = 5) were excluded from the final list. Dental infections (9/9; 100%) alongside SSTI (11/12; 84.6%) categories had the highest percentage of indicators that were rated appropriate and feasible with agreement. STIs (5/12; 55.6%), LUTIs (6/11; 54.5%) and general (4/8; 50%) categories had the lowest percentage of indicators rated appropriate and feasible with agreement.

## 3. Discussion

Our systematic RAM procedure developed a set of 61 quality indicators and general indicators that were rated both appropriate and feasible with agreement. This study, conducted as part of an international collaboration with SGUL, is an important step towards promoting the rational prescribing and use of antibiotics in PHC settings in South Africa. If antibiotic prescribing is inappropriate at the PHC level, this will continue to increase AMR at the population level, especially among LMICs [38,52]. Without evidence-based quality indicators, inappropriate use cannot be quantified and corrected. The final set comprises independent quality indicators that can be used to provide insight into the appropriateness of current and future antibiotic prescribing in primary care in South Africa in order to identify areas for intervention and improvement.

To the best of our knowledge, we believe this is the first study to assess a set of actionable AWaRe-based quality indicators for measuring the appropriateness of antibiotic prescribing specifically for the public primary care sector in South Africa, across multiple infection groups, using the RAM. Several studies have described various quality and prescribing indicators currently used in PHCs in South Africa [45,46,65,66,77,78]; however, the methodology used to develop them is often not fully described and some indicators focus more on antibiotic consumption rather than the appropriateness of prescribing. Most quality indicators have also been developed and applied in high-income countries [55,61,68,70,71,79,80,81], which are often not appropriate for LMICs with very different circumstances including often long waiting times and costs to see healthcare professionals in PHCs [20,67]. Alongside this, to date, evidence-based quality indicators have not been systematically developed and used for PHCs in LMIC settings, resulting in gaps in understanding the quality of antibiotic prescribing to facilitate tailored AMS interventions in primary care.

There is now generally international consensus on the treatment duration for common infections, contained in the WHO AWaRe book, replacing previous national guidelines where there have been issues [39,50]. Encouragingly, most treatment durations and doses for most infections in the South African Standard Treatment Guidelines (STGs) are generally aligned with the AWaRe antibiotic book [50,82,83]. During the rating process, South African STGs were considered where there are differences with the WHO AWaRe guidance [50,82,83]. For example, the first line treatment in primary care for bacterial eye infections is topical antibiotics; this resulted in the addition of two new statements during the second-round discussion to reflect the current standard treatment guidelines. The first line treatment for lower urinary tract infections is gentamicin injection; this resulted in rephrasing applicable statements to remove the word “oral” from the original statements. This was because the original statements were not considered appropriate for the South African context based on current treatment guidelines [82]. Since this study builds on the SGUL ADILA Quality Indicator studies and activities [54,66,75], the original statements that refer to the WHO AWaRe book were maintained for alignment purposes. Additionally, reference to the AWaRe book was maintained to allow for adaptability by other countries, especially other LMICs, with increasing use of the WHO AWaRe system to assess current prescribing practices as a prelude to instigating ASPs [67]. However, in practice, robust national treatment guidelines are usually prioritized considering local resistance patterns.

While prescribing in PHC facilities in South Africa, and among LMICs, is mainly undertaken by nurses [67,84,85,86], the panel emphasized that antibiotic prescribing should reflect evidence from the WHO AWaRe guidance and the current South African STGs for prescribing in primary care, regardless of who is prescribing or their professional qualification. Using these quality indicators in practice can help promote guideline adherence and evidence-based prescribing to reduce inappropriate antibiotic use while strengthening AMS.

The ratings focused on the average patient consulting at a PHC facility in South Africa; however, the panel discussions did not overlook patient variability and exceptions that may occur in practice. For example, intravenous antibiotics are generally not given for diarrhea in primary care. Consequently, malnourished and dehydrated pediatric patients who receive ceftriaxone for dysentery were considered an exception and not the average patient. In primary care, bacterial eye infections are generally treated with topical antibiotics. As a result, the use of ceftriaxone injection for neonates (neonatal conjunctivitis) when there is gonorrhea, syphilis or chlamydia, was considered an exception and not applicable to the average patient.

Where an indicator reflected inappropriate prescribing practice, e.g., the prescribing of an oral antibiotic for bronchitis or the proportion of patients receiving an antibiotic for acute infectious non-bloody diarrhea, the panel did not assign a low rating for appropriateness. While the practice is inappropriate, the indicator is considered an appropriate measure to assess the quality of care. Context specific considerations such as antibiotic resistance resulted in an indicator being rated lower for appropriateness but not feasibility. If data can be collected and the quality of care can be measured, then the indicator is feasible, even if it is considered inappropriate due to resistance issues, for example, URTIs which might be resistant to amoxicillin or other Access antibiotics. The panel also focused on resistance patterns that are expected in the community and not in a hospital setting. At a community level, the biases on resistance must be considered as well as the lack of data on resistance patterns for most pathogens.

While more than two thirds of the indicators were rated both feasible and appropriate with agreement, the current lack of electronic prescribing and electronic records in most provinces in South Africa negatively affected the feasibility of most of the indicators in public primary care settings. For example, facilities with no electronic systems may not be able to accurately quantify the use of antibiotics or accurately assess diagnosis-based indicators. In addition, while prescribing data can be collected from manual patient records in South African public PHCs, as previously undertaken with the APC-PPS [84], the process is labor intensive and time consuming. The adoption of electronic prescribing tools, supported by clinical decision support tools, is important to optimize antibiotic prescribing in primary care and mitigate challenges related to limited staff, time constraints, incomplete documentation and over reliance on paper-based processes [87,88,89]. Poor data availability and quality due to incomplete and inconsistent documentation in primary care records is a barrier that must be addressed going forward as this may well impede the feasibility of most indicators [90,91,92]. For example, all relevant information pertaining to an antibiotic allergy, i.e., severity, timing, and the nature of the allergy, as well as names of the antibiotics a patient is allergic to, may not be readily obtainable from the patient’s file. However, this is an important consideration before prescribing an antibiotic. Checking if the prescriber had previously documented if a patient has an allergy, including no allergy status, before prescribing an antibiotic could be more feasible with electronic systems in place compared with laboriously checking the relevant information pertaining to allergies in the patient’s file before prescribing an antibiotic.

For respiratory tract infections (RTIs), the panel identified the challenge of correctly defining bronchitis in primary care as a barrier to feasibility. Consequently, the clinical presentation for both suspected and confirmed infections for the average patient regardless of pathogen (viral or bacterial) must be considered. The lack of resistance data from the community for common RTIs also negatively impacts on the feasibility of the indicators. Whilst a cough can be a symptom of a non-respiratory infection tract illness, e.g., reflux, it was considered as the most likely symptom of a lower RTI for patients presenting at a primary care facility during the rating process. Consequently, this raises concerns about whether antibiotics are prescribed inappropriately.

Although all indicators for STIs were considered appropriate, they may not be feasible in primary care due to limited testing and diagnostic information. For these infections, i.e., chlamydia, gonorrhea and syphilis, diagnosis is syndromic and must be confirmed. Currently, it is more feasible to identify patients with syphilis compared to gonorrhea and chlamydia, as syphilis serology tests are sometimes carried out. In practice, prescribers do not distinguish between gonorrhea and chlamydia routinely. Additionally, the specific diagnosis is often not specified in the patient’s notes and is often generalized as genital ulcers/genital ulcer syndrome/genital discharge. Since these terms are not aligned with the WHO AWaRe book, the indicators in this category were not rephrased, as they would not be generalizable/applicable for the rest of Africa. This was identified as an area for quality improvement, especially if diagnostic tests that can differentiate between the different STIs are introduced in primary care, as opposed to quality assessment. Lack of laboratory facilities and diagnostic tests, laboratory capacity to identify causative organisms for infections, and their antimicrobial susceptibility in primary care, also contributes to AMR and needs to be urgently addressed going forward [93,94].

Our study has several strengths. We consider the evidence base of our study robust as we used model indicators developed using two discrete consensus technique methodologies [75]. The model indicators were firstly rated by a panel of more than 100 national experts in AMR and AMS from all the WHO regions, who rated the indicators in a country context using the Delphi technique, followed by a panel of 12 international leading experts in AMR/AMS, who rated the indicators in a global context using the RAM [75]. The additional indicators used in this study were based on the findings from a point prevalence survey in ambulatory care to better understand current presentation rates of clinical infections and antibiotic prescribing patterns for infections among public PHCs in South Africa [84], where these indicators will be used. Our expert panel was also multidisciplinary, involving a combination of national topic experts with international expertise and prescribers currently working in primary care. Alongside this, participation in the second round of ratings and the consensus meeting was high (83.3%), increasing the validity of the results. We employed the RAM, a common and validated consensus method, which has been utilized to develop quality indicators for antibiotic use in various settings worldwide [70,71,95,96]. The procedure we followed was consistent with the RAM guidelines [97], and the consensus meeting allowed the panel to discuss key challenges that may hinder the successful implementation of the indicators in practice. Subsequently, make recommendations that can improve the feasibility of the indicators in practice going forward. The indicators are based on the WHO AWaRe guidelines, which means our results can be generalized to a wider international population and the indicators can be applied in other LMICs, following the necessary context-specific adjustments and considerations.

However, we are aware that there are limitations to our study. While the RAM procedure was robust, the size of the panel may not fully reflect the diverse perspectives on antibiotic prescribing in primary care in South Africa. The perspectives of PHC prescribers from all South African provinces may also not have been fully captured, as the panel included only four PHC prescribers. The quality indicators were specifically rated for the public PHC sector context, and this may limit their applicability to the private PHC sector with different prescribing practices and less reliance on the local standard treatment guidelines. Despite these limitations, we believe the findings are robust, providing direction for the future to improve antibiotic prescribing in primary care in South Africa and wider areas.

Overall, we believe these indicated quality indicators can be adopted by the National Department of Health to form part of future quality assessments or clinical audits at PHC facilities, alongside any performance indicators currently in use. These audits are typically coordinated by PHC managers within their respective districts and undertaken at scheduled times during the year, ideally on a quarterly basis [98]. To address potential challenges with implementation capacity and human resources, we propose that high-priority indicators for infection categories where inappropriate prescribing is high, such as RTIs, for example, can be incorporated into future audits. We recommend a phased approach to implementing these indicators until electronic health records are fully implemented among PHC facilities in South Africa. Alongside this, there are dedicated AMS teams at PHC levels to facilitate routine assessment of antibiotic prescribing and propose future activities given current concerns with prescribing practices in South Africa [22,44,45,47,65]. These indicators can subsequently be used to assess the quality of prescribing both at the individual and facility levels. Where poor antibiotic prescribing practices are identified, appropriate quality improvement initiatives can be implemented in combination with educational programmes as part of agreed AMS activities [46,99]. Targeted educational interventions are seen as a cost-effective strategy to address inappropriate antibiotic prescribing among PHC prescribers, and we will be following this up in future research projects [52,100,101,102].

## 4. Materials and Methods

**Figure 1:**
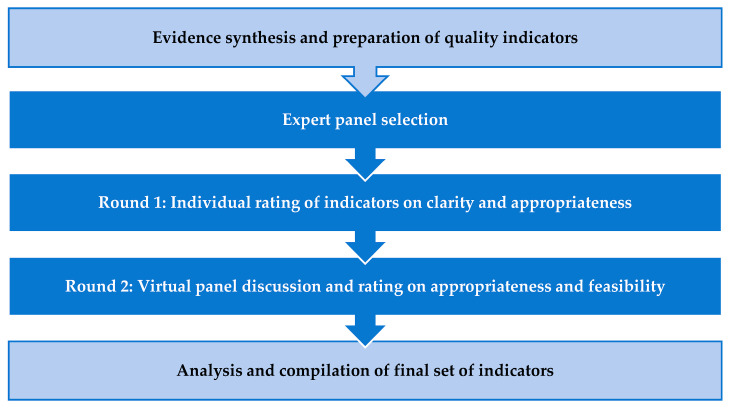
Overview of the RAM procedure.

The study used the RAM to develop quality indicators specific to public PHC settings in South Africa. The RAM, originally developed in the 1950s, is a commonly used consensus method which combines a synthesis of the evidence base with expert opinions through a two-round interactive process using questionnaires [70,71,97,103,104,105]. The technique facilitates the development of quality indicators that are evidence-based, clear, appropriate and feasible [105,106]. The process of identifying and selecting indicators using the RAM is outlined in Figure 1.

### 4.1. Evidence Synthesis and Preparation of Quality Indicators

The evidence for the quality indicators specific for use in assessing the appropriateness of antibiotic prescribing in primary care in South Africa was derived from three main sources:

(1) A published review of indicators for appropriate antibiotic use aligned to the WHO AWaRe system published in collaboration with SGUL, London, United Kingdom [54].
(2) Quality indicators and quantity metrics for optimal antibiotic use based on the WHO AWaRe system, developed as part of the global ‘Antimicrobial resistance, prescribing and consumption Data to Inform country antibiotic guidance and Local Action’ (ADILA) project [76]. The indicators were developed using two discrete consensus technique methodologies [75].
(3) Data from the Antibiotic Prescribing in Primary Healthcare Point Prevalence Survey (APC-PPS) conducted at eight PHC clinics in the North-West and Gauteng provinces in South Africa between November 2023 and October 2024, undertaken to better understand current presentation rates of clinical infections and antibiotic prescribing patterns for infections at PHCs [83,107]. The results of the APC-PPS showed that acute cough and sexually transmitted infections were the most reported infection presentations at PHCs [93]. This resulted in the development of additional indicators to measure the appropriateness of antibiotic prescribing for acute cough and sexually transmitted infections using guidance from the AWaRe Book [50].

A total of 78 quality indicators derived from the three sources were categorized into eight infection areas. Sixty-three indicators were identified from the SGUL indicators, 14 indicators were identified from the PPS, and one indicator was identified from the literature [60,85,95]. The key terms, rating scales and the interpretation of the levels of appropriateness and feasibility for this study were defined in preparation for the two rounds of rating (Table 4).

### 4.2. Expert Panel Selection and Composition

The selection of the expert panel was based on the RAM guidelines [97]. It was important to balance representation from national topic specialists and prescribers currently working in public primary care, where the indicators would be used. The 12-member panel comprised seven national experts in infectious diseases, medical microbiology, antimicrobial stewardship, public health, clinical pharmacy, quality improvement, academia and healthcare policy development and coordination; two medical officers and two professional nurses working in primary care and one professional nurse working for the National Department of Health. A panel size of 12 experts was considered ideal for a balanced multidisciplinary panel and to ensure at least 9 members participated in the second round. The experts were identified and recruited based on relevant professional knowledge and expertise, geographic and professional diversity, absence of any conflicts of interest that could bias ratings, and willingness to participate in the full RAM process. Experts who had previously represented South Africa in the global Delphi panel led by the SGUL team were invited to participate.

### 4.3. Round 1: Rating Clarity and Appropriateness

The first-round rating took place in August 2025. In this round, panelists were requested to independently and confidentially rate the clarity and appropriateness of each of the 78 candidate quality indicators on separate 9-point Likert Scales. The list of indicators, organized into eight separate tabs on a Microsoft 365 Excel™ spreadsheet, was sent via email for each panel member to rate and submit within 14 calendar days. For each indicator, the source of the indicator was cited at the end of each row, in the final column of the respective tabs. Each tab had a section for rephrasing statements, new suggested statements and comments. The spreadsheet also included tabs with rating instructions, rating scales and references. An evidence summary and a guidance document were also attached separately in the email sent to the panelists.

### 4.4. Round 2: Consensus Meeting and Rating Appropriateness and Feasibility

The virtual interactive meeting and second round rating took place in September 2025 on Microsoft 365 Teams™. A week before the virtual meeting, the rating sheet and scales for the second round, rating instructions, individual feedback from the first round, the overall panel median and the frequency distribution of panel ratings were emailed to each panel member in preparation for the meeting. The results from the first round were also shared with the meeting co-chairs (MM and SMC).

The three-hour consensus meeting was co-chaired and moderated by a RAM expert (SMC) and a topic specialist (MM) to discuss the ratings of the first round and re-rate each statement separately on the appropriateness and feasibility scales. No indicators were discarded from the first round. Suggestions for rephrasing were made for nine statements in the first round for rating in the second round. Two new statements were proposed during the meeting. As a result, 89 statements were rated for appropriateness and feasibility in the second round. Quality indicators that did not reach consensus in the first round were discussed during the meeting before they were rated again.

### 4.5. Data Analysis

Data from both rounds were analyzed using Microsoft 365 Excel™. The median ratings and level of consensus were calculated for each indicator for clarity and appropriateness in the first round and for appropriateness and feasibility in the second round. The criteria to determine the three levels of consensus (agreement, equivocal and disagreement) are presented in Table 1. The quality indicators rated as both appropriate and feasible with agreement in the second round were included in the final set of quality indicators. In this paper, only the results from the second round are presented.

## 5. Conclusions

This study used a systematic and validated approach to develop a set of 61 appropriate and feasible quality indicators, combining evidence and expert opinion. These AWaRe-based indicators can be used for monitoring antibiotic prescribing and use in PHC settings in South Africa and can be adapted by other LMICs. We are currently testing the applicability of a subset of the indicators, to establish their clinimetric properties in South African public PHC settings. This is important to externally validate the indicators, beyond the panel’s consensus and to confirm the feasibility and effectiveness of the indicators in clinical settings. The findings of this study are important for antimicrobial stewardship and quality improvement in primary care.

## Figures and Tables

**Table 1:** Summary of median scores and level of consensus of Round 2 appropriateness and feasibility ratings.

	Infection Categories								
	RTI	LUTI	SSTI	Diarrhea & Enteric Fever	STI	Bacterial Eye	Dental	General	Total
Indicators (*n*)	19	11	13	9	12	8	9	8	89
Appropriateness: Number (%) of indicators per median score									
Median 7–9 (appropriate)	19 (100%)	11 (100%)	13 (100%)	9 (100%)	12 (100%)	8 (100%)	9 (100%)	8 (100%)	89 (100%)
Appropriateness: Number (%) of indicators per level of consensus									
Agreement	16 (84.2%)	9 (81.8%)	11 (84.6%)	7 (77.8%)	12 (100%)	5 (62.5%)	9 (100%)	9 (100%)	77 (86.5%)
Equivocal	3 (15.8%)	2 (18.2%)	2 (15.4%)	2 (22.2%)	0	3 (37.5%)	0	0	12 (13.5%)
Feasibility: Number (%) of indicators per median score									
Median 4–6 (equivocal)	0	0	0	0	2 (16.7%)	0	0	2 (25%)	4 (4.5%)
Median 7–9 (feasible)	19 (100%)	11 (100%)	13 (100%)	9 (100%)	10 (83.3%)	8 (100%)	9 (100%)	6 (75%)	85 (95.5%)
Feasibility: Number (%) of indicators per level of consensus									
Agreement	17 (89.5%)	10 (90.9%)	13 (100%)	6 (66.7%)	9 (75%)	7 (87.5%)	9 (100%)	4 (50%)	75 (84.3%)
Equivocal	2 (10.5%)	1 (9.1%)	0	3 (33.3%)	3 (25%)	1 (12.5%)	0	4 (50%)	14 (15.7%)

LUTI = lower urinary tract infection; RTI = respiratory tract infection; SSTI = skin and soft tissue infection; STI = sexually transmitted infection.

**Table 2:** Round 2 outcomes of indicators rated for appropriateness and feasibility (*n* = 89).

Infection (Number of Indicators)	No.	Statement	Appropriateness			Feasibility		
			Med	LOC	A (%) ^҂^	Med	LOC	A (%) ^҂^
RTI (*n* = 19)	1	Proportion of all patients presenting with any acute RTI given an oral antibiotic	9	E	60%	8	A	100%
	2	Proportion of all patients presenting with any acute RTI given oral amoxicillin	8	A	100%	8	A	100%
	3	Proportion of all patients presenting with any acute RTI given any oral Access antibiotic (including amoxicillin)	8	A	100%	8	A	100%
	4	Proportion of all patients presenting with any acute RTI given any oral Watch antibiotic	8	A	100%	7	A	100%
	5	Proportion of patients presenting with bronchitis given an oral antibiotic	8	A	100%	7	E	50%
	6	Proportion of patients with any ear/sinus/throat infection (not pneumonia) given an oral antibiotic	8	A	100%	8	A	100%
	7	Proportion of patients with any ear/sinus/throat infection (not pneumonia) at high risk * of severe complications given an oral antibiotic	8	A	100%	8	E	70%
	8	Proportion of patients with any ear/sinus/throat infection (not pneumonia) at high risk * of severe complications given amoxicillin	8	A	100%	8	A	80%
	9	Proportion of patients with any ear/sinus/throat infection (not pneumonia) at high risk * of severe complications given any oral Access antibiotic (including amoxicillin)	8	A	100%	8	A	100%
	10	Proportion of patients at lower risk of a bacterial respiratory tract infection given an oral antibiotic	8	A	100%	7	A	80%
	11	Proportion of patients with acute RTIs given the duration in days of oral antibiotics recommended in the WHO AWaRe Antibiotic Book	8	A	100%	8	A	100%
	12	Proportion of patients with acute RTIs prescribed the total daily dose of oral antibiotics recommended in the WHO AWaRe Antibiotic Book	9	E	60%	7	A	90%
	13	Proportion of patients with bacterial RTIs given oral Access or Watch antibiotics	8	A	100%	7	A	90%
	14	Proportion of patients (no relevant comorbidities) presenting with acute cough who should be prescribed oral antibiotics ^‡^	7	A	90%	7	A	80%
	14a	Proportion of patients (no relevant comorbidities) presenting with acute cough, who met WHO AWaRe guidelines for antibiotic prescription ^†^	8	A	100%	7	A	80%
	15	Proportion of patients (no relevant comorbidities) presenting with acute cough prescribed Access antibiotics	8	A	100%	8	A	100%
	16	Proportion of patients (no relevant comorbidities) presenting with acute cough prescribed Watch antibiotics	8	A	100%	8	A	100%
	17	Proportion of acute cough cases with documented bacterial indications (clinical justification for antibiotic use where the documented signs are suggestive of bacterial infection, e.g., fever > 38 °C, purulent sputum, dyspnea, or suspected pneumonia)	9	A	90%	8	A	90%
	18	Proportion of antibiotics prescribed for acute cough that are aligned with treatment guidelines (e.g., acute cough if pneumonia is suspected)	7	E	60%	8	A	100%
LUTI (*n* = 11)	19	Proportion of patients presenting with lower urinary tract infection (UTI) given an oral antibiotic ^‡^	8	A	80%	8	A	100%
	19a	Proportion of patients presenting with LUTI given an antibiotic ^†^	9	A	90%	8	A	100%
	20	Proportion of low-risk * patients with positive urine test (positive urine leucocytes/leucocyte esterase or positive urine culture), but no UTI symptoms (e.g., no dysuria, no increased urinary urgency and frequency, no lower abdominal pain or discomfort or sometimes visible hematuria), given oral antibiotics ^‡^	8	E	70%	8	E	70%
	20a	Proportion of low-risk * patients with positive urine test (positive urine leucocytes/leucocyte esterase or positive urine culture), but no UTI symptoms (e.g., no dysuria, no increased urinary urgency and frequency, no lower abdominal pain or discomfort or sometimes visible hematuria), given antibiotics ^†^	9	A	90%	8	A	90%
	21	Proportion of patients presenting with LUTI given any oral Access antibiotic ^‡^	8	A	80%	8	A	100%
	21a	Proportion of patients presenting with LUTI given any Access antibiotic ^†^	8	A	100%	8	A	100%
	22	Proportion of patients with LUTI given oral Watch antibiotics ^₶^	8	A	90%	8	A	100%
	23	Proportion of patients presenting with LUTI given any oral Watch antibiotic ^₶^	8	A	90%	8	A	90%
	24	Proportion of patients presenting with LUTIs given the duration in days of oral antibiotics recommended in the WHO AWaRe Antibiotic Book ^‡^	9	E	60%	8	A	100%
	24a	Proportion of patients presenting with LUTIs given the duration in days of antibiotics recommended in the WHO AWaRe Antibiotic Book ^†^	9	A	100%	8	A	100%
	25	Proportion of patients presenting with LUTI prescribed the total daily dose of oral antibiotics recommended in the WHO AWaRe Antibiotic Book	8	A	90%	8	A	100%
SSTI (*n* = 13)	26	Proportion of patients presenting with acute lymphadenitis given an oral antibiotic	8	A	100%	8	A	90%
	27	Proportion of patients presenting with higher risk * (of bacterial infection) acute lymphadenitis given an oral antibiotic	9	E	60%	7	A	90%
	28	Proportion of patients presenting with higher risk * (of bacterial infection) acute lymphadenitis given any oral Access antibiotic	9	A	80%	7	A	90%
	29	Proportion of patients presenting with higher risk * (of bacterial infection) acute lymphadenitis given any oral Watch antibiotic	8	A	100%	7	A	80%
	30	Proportion of patients with lower risk (of bacterial infection) acute lymphadenitis given oral antibiotics	8	A	90%	7	A	90%
	31	Proportion of patients presenting with acute lymphadenitis given the duration in days of oral antibiotics recommended in the WHO AWaRe Antibiotic Book	9	A	90%	8	A	80%
	32	Proportion of patients presenting with acute lymphadenitis prescribed the total daily dose of oral antibiotics recommended in the WHO AWaRe Antibiotic Book	9	A	90%	8	A	80%
	33	Proportion of patients presenting with mild ** SSTIs given an oral antibiotic	8	A	100%	7	A	90%
	34	Proportion of patients presenting with mild ** SSTIs given a topical antibiotic	8	A	90%	8	A	90%
	35	Proportion of patients presenting with mild ** SSTIs given any oral Access antibiotic	8	A	90%	7	A	90%
	36	Proportion of patients presenting with mild ** SSTIs given any oral Watch antibiotic	9	E	70%	7	A	90%
	37	Proportion of patients presenting with mild ** SSTIs given the duration in days of oral antibiotics recommended in the WHO AWaRe Antibiotic Book	9	A	90%	8	A	90%
	38	Proportion of patients presenting with mild ** SSTIs prescribed the total daily dose of oral antibiotics recommended in the WHO AWaRe Antibiotic Book	8	A	90%	8	A	90%
Diarrhea & enteric fever (*n* = 9)	39	Proportion of all patients presenting with high-risk * acute infectious non-bloody diarrhea given any oral Watch antibiotic	8	A	100%	7	A	90%
	40	Proportion of all patients presenting with acute infectious non-bloody diarrhea given oral antibiotics	9	E	70%	8	E	70%
	41	Proportion of all patients presenting with high-risk * acute infectious non-bloody diarrhea given any oral Access antibiotic	9	E	70%	7	A	90%
	42	Proportion of otherwise healthy patients presenting with acute infectious non-bloody diarrhea given an oral antibiotic	9	A	80%	7	A	90%
	43	Proportion of patients presenting with high-risk * acute infectious non-bloody diarrhea given the duration in days of oral antibiotics recommended in the WHO AWaRe Antibiotic Book	8	A	90%	7	A	90%
	44	Proportion of patients presenting with high-risk * acute infectious non-bloody diarrhea prescribed the total daily dose of oral antibiotics recommended in the WHO AWaRe Antibiotic Book	8	A	90%	7	A	90%
	45	Proportion of patients with acute bloody infectious diarrhea given oral Access or Watch antibiotics	8	A	100%	8	A	80%
	46	Proportion of patients with acute bloody infectious diarrhea given oral Access antibiotics	9	A	90%	8	E	70%
	47	Proportion of patients with acute bloody infectious diarrhea given oral Watch antibiotics	9	A	80%	8	E	70%
STI (*n* = 12)	48	Proportion of patients (adults and young people aged over 12 years) presenting with uncomplicated chlamydial urogenital infection (not pregnant) treated with oral Access antibiotics	9	A	90%	6	E	50%
	49	Proportion of patients (adults and young people aged over 12 years) presenting with uncomplicated chlamydial urogenital infection (not pregnant) treated with oral Watch antibiotics	9	A	90%	6	E	50%
	50	Proportion of patients (adults and young people aged over 12 years) presenting with uncomplicated chlamydial urogenital infection prescribed the doses and durations for antibiotics recommended in the WHO AWaRe Antibiotic Book ^‡^	8	A	100%	7	A	90%
	50a	Proportion of patients (adults and young people aged over 12 years) presenting with uncomplicated chlamydial urogenital infection prescribed the doses for antibiotics recommended in the WHO AWaRe Antibiotic Book ^†^	9	A	80%	7	A	90%
	50b	Proportion of patients (adults and young people aged over 12 years) presenting with uncomplicated chlamydial urogenital infection prescribed the durations for antibiotics recommended in the WHO AWaRe Antibiotic Book ^†^	9	A	80%	7	A	90%
	51	Proportion of patients (adults and young people aged over 12 years) presenting with genital, anorectal or oropharyngeal gonococcal infection who are treated with first line single therapy	9	A	80%	7	A	80%
	52	Proportion of patients (adults and young people aged over 12 years) presenting with gonococcal infection who are treated with first line dual therapy	9	A	90%	7	E	70%
	53	Proportion of patients (adults and young people aged over 12 years) presenting with gonococcal infection who are retreated if symptoms persist after 5 days of adequate treatment	9	A	80%	7	A	80%
	54	Proportion of patients (adults and young people aged over 12 years) presenting with gonococcal infection who are retreated with Access antibiotics if symptoms persist after 5 days of adequate treatment	8	A	100%	7	A	90%
	55	Proportion of patients (adults and young people aged over 12 years) presenting with early or late syphilis treated with first-line Access antibiotics	8	A	100%	8	A	100%
	56	Proportion of patients (adults and young people aged over 12 years) presenting with early or late syphilis treated with any Watch antibiotics	8	A	100%	7	A	90%
	57	Proportion of patients presenting with genital discharge/STI prescribed antibiotics with complete documentation of symptoms, history, diagnosis, tests, etc.	8	A	100%	7	A	90%
Bacterial eye (*n* = 8)	58	Proportion of patients presenting with eye infection given an oral antibiotic	8	E	70%	7	E	60%
	58a	Proportion of patients with eye infections prescribed topical antibiotics recommended in the WHO AWaRe Antibiotic Book ^⮾^	8	A	100%	8	A	90%
	58b	Proportion of patients with bacterial eye infections prescribed topical antibiotics recommended in the WHO AWaRe Antibiotic Book ^⮾^	9	A	90%	8	A	80%
	59	Proportion of patients presenting with bacterial eye infections given the duration in days of oral antibiotics recommended in the WHO AWaRe Antibiotic Book	8	A	100%	8	A	90%
	60	Proportion of patients with bacterial eye infections prescribed the total daily dose of oral antibiotics recommended in the WHO AWaRe Antibiotic Book	8	A	100%	8	A	90%
	61	Proportion of patients with eye infections prescribed the total daily dose of oral antibiotics recommended in the WHO AWaRe Antibiotic Book	7	A	90%	8	A	90%
	62	Proportion of patients presenting with eye infection at high risk * of severe complications given any oral Access antibiotic	9	E	60%	7	A	90%
	63	Proportion of patients presenting with eye infection at high risk * of severe complications given any oral Watch antibiotic	9	E	70%	7	A	90%
Dental (*n* = 9)	64	Proportion of otherwise healthy adults presenting with dental infections given an oral antibiotic	8	A	100%	8	A	90%
	65	Proportion of otherwise healthy adults presenting with severe ** dental infections given an oral antibiotic	9	A	90%	8	A	90%
	66	Proportion of patients presenting with dental infections at high risk * of severe complications given an oral antibiotic	8	A	100%	7	A	90%
	67	Proportion of patients presenting with dental infections at high risk * of severe complications given amoxicillin	8	A	100%	7	A	90%
	68	Proportion of patients presenting with dental infections at high risk * of severe complications given any oral Access antibiotic (including amoxicillin)	8	A	90%	7	A	80%
	69	Proportion of patients with lower risk (of bacterial infection) dental infections given oral antibiotics	8	A	100%	7	A	90%
	70	Proportion of patients presenting with dental infections given the duration in days of oral antibiotics recommended in the WHO AWaRe Antibiotic Book	8	A	90%	8	A	100%
	71	Proportion of patients with dental infections prescribed the total daily dose of oral antibiotic recommended in the WHO AWaRe Antibiotic Book	8	A	100%	8	A	100%
	72	Proportion of patients with dental infections given oral Watch antibiotics	8	A	100%	8	A	100%
General (*n* = 8)	73	Allergy status of the patient including timing, nature and severity of previous exposure/possible allergic reactions to antibiotics and the name/s of the antibiotic should be documented in the medical records ^‡^	9	A	90%	5	E	50%
	73a	Allergy status of the patient to any antibiotic should be documented in the medical records ^†^	9	A	100%	8	A	100%
	74	Any toxicity/adverse reaction to an antibiotic (including type, duration of symptoms, time of onset since antibiotic administration) should be documented in medical records	9	A	100%	5	E	40%
	75	Use of oral Access and Watch antibiotics (split by AWaRe group) measured in DID in primary care	9	A	90%	7	E	70%
	76	At least 80% of total oral antibiotic use in primary care should be Access antibiotics	9	A	90%	8	A	100%
	77	Percentage of total oral Access antibiotic use	9	A	90%	8	A	90%
	78	Ratio of oral amoxicillin measured in DID in primary care to all oral antibiotics in primary care measured in DID excluding amoxicillin (including amoxicillin-clavulanic acid) ^‡^	8	A	100%	8	A	90%
	78a	Ratio of oral amoxicillin measured in DID in primary care to all other oral antibiotics in primary care measured in DID excluding amoxicillin (including amoxicillin-clavulanic acid) ^†^	8	A	100%	7	E	70%

A = agreement; DID = Defined daily doses per 1000 inhabitants per day; E = equivocal; LOC = level of consensus; LUTI = lower urinary tract infection; Med = median; RTI = respiratory tract infection; SSTI = skin and soft tissue infection; STI = sexually transmitted infection; * = as per AWaRe guidance [42,55]; ** = severity criteria as per AWaRe guidance; ^҂^ agreement = rated within ±1 of the median; ^‡^ = original statement; ^†^ = rephrased statement; ^₶^ = identified and rated as similar statements; ^⮾^ = new statement.

**Table 3:** Set of indicators rated appropriate and feasible in Round 2 (*n* = 61; *N* = 89).

Infection (*n*/*N*; %)	No.	Statement
RTI (13/19; 68.4%)	1.	Proportion of all patients presenting with any acute RTI given oral amoxicillin
	2.	Proportion of all patients presenting with any acute RTI given any oral Access antibiotic including amoxicillin
	3.	Proportion of all patients presenting with any acute respiratory tract infection (RTI) given any oral Watch antibiotic
	4.	Proportion of patients with any ear/sinus/throat infection (not pneumonia) given an oral antibiotic
	5.	Proportion of patients with any ear/sinus/throat infection (not pneumonia) at high risk * of severe complications given amoxicillin
	6.	Proportion of patients with any ear/sinus/throat infection (not pneumonia) at high risk * of severe complications given any oral Access antibiotic (including amoxicillin)
	7.	Proportion of patients at lower risk of a bacterial respiratory tract infection given an oral antibiotic
	8.	Proportion of patients with acute RTIs given the duration in days of oral antibiotics recommended in the WHO AWaRe Antibiotic Book
	9.	Proportion of patients with bacterial RTIs given oral Access or Watch antibiotics
	10.	Proportion of patients (no relevant comorbidities) presenting with acute cough, who met WHO AWaRe guidelines for antibiotic prescription
	11.	Proportion of patients (no relevant comorbidities) presenting with acute cough prescribed Access antibiotics
	12.	Proportion of patients (no relevant comorbidities) presenting with acute cough prescribed Watch antibiotics
	13.	Proportion of acute cough cases with documented bacterial indications (clinical justification for antibiotic use where the documented signs are suggestive of bacterial infection, e.g., fever > 38 °C, purulent sputum, dyspnea, or suspected pneumonia)
LUTI (6/11; 54.5%)	14.	Proportion of patients presenting with LUTI given an antibiotic.
	15.	Proportion of low-risk patients * with positive urine test (positive urine leucocytes/leucocyte esterase or positive urine culture), but no UTI symptoms (e.g., no dysuria, no increased urinary urgency and frequency, no lower abdominal pain or discomfort or sometimes visible hematuria), given antibiotics
	16.	Proportion of patients presenting with LUTI given any Access antibiotic
	17.	Proportion of patients with LUTI given oral Watch antibiotics
	18.	Proportion of patients presenting with LUTIs given the duration in days of antibiotics recommended in the WHO AWaRe Antibiotic Book
	19.	Proportion of patients presenting with LUTI prescribed the total daily dose of oral antibiotics recommended in the WHO AWaRe Antibiotic Book
SSTI (11/13; 84.6%)	20.	Proportion of patients presenting with acute lymphadenitis given an oral antibiotic
	21.	Proportion of patients presenting with higher risk * (of bacterial infection) acute lymphadenitis given any oral Access antibiotic
	22.	Proportion of patients presenting with higher risk * (of bacterial infection) acute lymphadenitis given any oral Watch antibiotic
	23.	Proportion of patients with lower risk (of bacterial infection) acute lymphadenitis given oral antibiotics
	24.	Proportion of patients presenting with acute lymphadenitis given the duration in days of oral antibiotics recommended in the WHO AWaRe Antibiotic Book
	25.	Proportion of patients presenting with acute lymphadenitis prescribed the total daily dose of oral antibiotics recommended in the WHO AWaRe Antibiotic Book
	26.	Proportion of patients presenting with mild * SSTIs given an oral antibiotic
	27.	Proportion of patients presenting with mild * SSTIs given a topical antibiotic
	28.	Proportion of patients presenting with mild * SSTIs given any oral Access antibiotic
	29.	Proportion of patients presenting with mild * SSTIs given the duration in days of oral antibiotics recommended in the WHO AWaRe Antibiotic Book
	30.	Proportion of patients presenting with mild * SSTIs prescribed the total daily dose of oral antibiotics recommended in the WHO AWaRe Antibiotic Book
Diarrhea & enteric fever (5/9; 55.6%)	31.	Proportion of all patients presenting with high-risk * acute infectious non-bloody diarrhea given any oral Watch antibiotic
	32.	Proportion of otherwise healthy patients presenting with acute infectious non-bloody diarrhea given an oral antibiotic
	33.	Proportion of patients presenting with high-risk * acute infectious non-bloody diarrhea given the duration in days of oral antibiotics recommended in the WHO AWaRe Antibiotic Book
	34.	Proportion of patients presenting with high-risk * acute infectious non-bloody diarrhea prescribed the total daily dose of oral antibiotics recommended in the WHO AWaRe Antibiotic Book
	35.	Proportion of patients with acute bloody infectious diarrhea given oral Access or Watch antibiotics
STI (8/12; 66.7%)	36.	Proportion of patients (adults and young people aged over 12 years) presenting with uncomplicated chlamydial urogenital infection prescribed the doses for antibiotics recommended in the WHO AWaRe Antibiotic Book
	37.	Proportion of patients (adults and young people aged over 12 years) presenting with uncomplicated chlamydial urogenital infection prescribed the durations for antibiotics recommended in the WHO AWaRe Antibiotic Book
	38.	Proportion of patients (adults and young people aged over 12 years) presenting with genital, anorectal or oropharyngeal gonococcal infection who are treated with first line single therapy
	39.	Proportion of patients (adults and young people aged over 12 years) presenting with gonococcal infection who are retreated if symptoms persist after 5 days of adequate treatment
	40.	Proportion of patients (adults and young people aged over 12 years) presenting with gonococcal infection who are retreated with Access antibiotics if symptoms persist after 5 days of adequate treatment
	41.	Proportion of patients (adults and young people aged over 12 years) presenting with early or late syphilis treated with first line Access antibiotics
	42.	Proportion of patients (adults and young people aged over 12 years) presenting with early or late syphilis treated with any Watch antibiotics
	43.	Proportion of patients presenting with genital discharge/STI prescribed antibiotics with complete documentation of symptoms, history, diagnosis, tests, etc.
Bacterial eye (5/8; 62.5%)	44.	Proportion of patients presenting with bacterial eye infections given the duration in days of oral antibiotics recommended in the WHO AWaRe Antibiotic Book
	45.	Proportion of patients with bacterial eye infections prescribed the total daily dose of oral antibiotics recommended in the WHO AWaRe Antibiotic Book
	46.	Proportion of patients with eye infections prescribed the total daily dose of oral antibiotics recommended in the WHO AWaRe Antibiotic Book
	47.	Proportion of patients with eye infections prescribed topical antibiotics recommended in the WHO AWaRe Antibiotic Book
	48.	Proportion of patients with bacterial eye infections prescribed topical antibiotics recommended in the WHO AWaRe Antibiotic Book
Dental (9/9; 100%)	49.	Proportion of otherwise healthy adults presenting with dental infections given an oral antibiotic
	50.	Proportion of otherwise healthy adults presenting with severe ** dental infections given an oral antibiotic
	51.	Proportion of patients presenting with dental infections at high risk * of severe complications given an oral antibiotic
	52.	Proportion of patients presenting with dental infections at high risk * of severe complications given amoxicillin
	53.	Proportion of patients presenting with dental infections at high risk * of severe complications given any oral Access antibiotic (including amoxicillin)
	54.	Proportion of patients with lower risk (of bacterial infection) dental infections given oral antibiotics
	55.	Proportion of patients presenting with dental infections given the duration in days of oral antibiotics recommended in the WHO AWaRe Antibiotic Book
	56.	Proportion of patients with dental infections prescribed the total daily dose of oral antibiotic recommended in the WHO AWaRe Antibiotic Book
	57.	Proportion of patients with dental infections given oral Watch antibiotics
General (4/8; 50%)	58.	Allergy status of the patient to any antibiotic should be documented in the medical records
	59.	At least 80% of total oral antibiotic use in primary care should be Access antibiotics
	60.	Percentage of total oral Access antibiotic use
	61.	Ratio of oral amoxicillin measured in DID in primary care to all oral antibiotics in primary care measured in DID, excluding amoxicillin (including amoxicillin-clavulanic acid)

DID = Defined daily doses per 1000 inhabitants per day; LUTI = lower urinary tract infection; *n* = number of indicators rated appropriate and feasible with agreement; *N* = number of indicators rated in Round 2; RTI = respiratory tract infection; SSTI = skin and soft tissue infection; STI = sexually transmitted infection; * = as per AWaRe guidance [42,55]; ** = severity as per AWaRe guidance.

**Table 4:** Definitions and rating scales.

	Definitions
Key Terms	
Appropriateness	The extent to which an indicator is beneficial, effective, and evidence-based (or clinically indicated) when applied to the ‘average’ patient in primary care
Feasibility	The practicality of implementing the indicator in routine clinical practice (i.e., is it feasible from a human, data, workforce and financial perspective in the South African context?)
Clarity	The degree to which the indicator is clearly and precisely defined, unambiguous, and easily understood by clinicians and stakeholders
Rating scores for appropriateness and feasibility (9-point Likert scale)	
1	Completely inappropriate/infeasible (no exceptions ever)
2	Very inappropriate/infeasible: rare exceptions
3	Inappropriate/infeasible: some exceptions
4	Equivocal but inappropriate/infeasible for many
5	Equivocal
6	Equivocal but appropriate/feasible for some
7	Appropriate/feasible: some exceptions
8	Very appropriate/feasible: rare exceptions
9	Completely appropriate/feasible (no exceptions ever)
Levels of appropriateness and feasibility	
1–3	Inappropriate/infeasible
4–6	Equivocal
7–9	Appropriate/feasible
Levels of consensus	
Agreement (A)	≥80% rated within ±1 of the median
Disagreement (D)	≥33% rated in both 1–3 and 7–9 score ranges
Equivocal (E)	Neither disagreement nor agreement (no consensus)

## Data Availability

Additional data is available from the corresponding authors on reasonable request.

## Supplementary Materials

The following supporting information can be downloaded at: https://www.mdpi.com/article/10.3390/antibiotics15020196/s1. Table S1: Current antibiotic prescribing concerns in primary care in South Africa. References [41,42,44,45,46,47,48,65,77,84,86,93,108] are cited in the Supplementary Materials.
