# Supplementary material for: Development of AWaRe-Based Quality Indicators to Assess the Appropriateness of Antibiotic Prescribing in Primary Healthcare in South Africa
Source: Antibiotics (Basel). 2026 Feb 10;15(2):196. doi: 10.3390/antibiotics15020196 (PMC12937462; doi:10.3390/antibiotics15020196)
Supplement: Supplementary file 1 [file antibiotics-15-00196-s001.zip › antibiotics-4085536-supplementary.pdf]

Supplementary Table S1 – Current antibiotic prescribing concerns in primary care in South Africa

Author and year	Aim and methods	Summary of key findings
Farley et al., 2018 [41]	• Cross sectional survey design with a self-administered questionnaire to assess attitudes, knowledge and practices regarding antibiotics and ABR among primary care prescribers<br>• 264 prescribers completed the survey, 98.3% were physicians and 84.8% were practising in the private sector	• 95.8% of prescribers viewed ABR as a major challenge in South Africa<br>• 87.5% of prescribers indicated a need for further education on the appropriate use of antibiotics in primary care, and 96.2% expressed a desire for access to local ABR data to guide future prescribing<br>• Prescribers were also interested in the provision of STGs in different formats to improve future prescribing<br>• However, 66.5% of prescribers reported feeling pressured by patients to prescribe antibiotics for their presenting infectious illness, regardless of whether the antibiotics were needed
Gasson et al., 2018 [65]	• Antibiotic prescribing habits were assessed retrospectively among prescribers in 8 PHCs alongside potential reasons for non-adherence to STGs<br>• 654 patient records were reviewed	• 68.7% of patients attending these PHCs were prescribed an antibiotic<br>• There were concerns with low adherence to STGs as adherence only occurred in 45.1% of prescriptions<br>• The main reasons for non-adherence to STGs included: undocumented diagnoses (30.5%), antibiotics not required including for self-limiting viral infections (21.6%), incorrect doses of antibiotics prescribed (12.9%), incorrect duration of antibiotic therapy prescribed (9.5%), and incorrect treatment for the presenting infectious disease (1.5%)
Truter and Knoesen, 2018 [42]	• Study used a self-administered questionnaire to determine antibiotic prescribing habits among primary care physicians<br>• 16 community pharmacists in the Eastern Cape province participated in the study	• 81.3% of participants believed physicians over-prescribed antibiotics, including for viral infections, influenced by patient pressure<br>• Amoxicillin/co-amoxiclav were the most prescribed antibiotics, followed by clarithromycin, ciprofloxacin and azithromycin<br>• Surveyed community pharmacists believed sinusitis and URTIs were the most common infections for which antibiotics were prescribed
van Hecke et al., 2019 [93]	• The objective was to determine the perceptions of clinicians working in PHCs concerning antibiotic prescribing for acute coughs and UTIs alongside their experiences concerning point-of-care testing<br>• Qualitative interviews were undertaken among 23 prescribers	• Antibiotic prescribing decisions among participating HCPs in PHCs were typically influenced by a number of factors, including their clinical assessment of presenting patients, their comorbidities and perceptions regarding patient expectations<br>• There were observed difficulties in the communication between prescribers and patients, which often hampered efforts to explain non-antibiotic management strategies to patients including for viral infections<br>• In view of this, participating clinicians were typically positive towards current and future point-of-care testing, especially for viral infections, to improve evidence-based antibiotic prescribing<br>• Prescribers though had concerns with current resources and workflow issues influencing the uptake and use of

Author and year	Aim and methods	Summary of key findings
		such tests as part of their routine care of patients presenting with infectious diseases
Govender et al., 2021 [44]	• The study aimed to evaluate the use and implementation of the STGs/EML among 98 prescribers (nurses) at a public tertiary institution and associated PHC facilities<br>• A mixed approach was used. This included evaluating patient records and undertaking interviews using a structured questionnaire	• Only 41.8% of nurses had access to the latest STGs/EML<br>• All the participating nurses stated they often/ sometimes refer to the STGs/EML when managing patients.<br>• 78.3% of prescriptions had the diagnosis recorded, with a 59.7% adherence rate to the STGs/EML.<br>• Most nurses (94.9%) requested training on the use of STGs/EML to improve future antibiotic prescribing
Alabi et al, 2022 [45]	• The objective was to assess the appropriateness of antibiotic prescribing among practicing GPs in the private sector<br>• This included an analysis of antibiotic prescriptions (188,141) among 174,889 patients, with appropriateness based on ICD-10 classification and whether an antibiotic was deemed warranted or not	• 92.9% of patients were prescribed one antibiotic by primary care GPs when attending their clinics, with 7.1% prescribed two or more antibiotics<br>• Penicillins were the most prescribed antibiotics (40.7%) of all antibiotics prescribed. This was followed by macrolides (16.8%) and cephalosporins (15.7% - all generations combined)<br>• 46.1% of all diagnoses made included diseases of the respiratory system<br>• 8.8% of all the prescriptions were subsequently deemed as appropriate and 32.0% potentially appropriate. However, 45.4% of prescriptions were seen as inappropriate and 13.8% could not be assessed due to a lack of specific codes/ contained unlisted codes/ or contained unclear descriptions in the prescription
De Vries et al., 2022 [46]	• The objective was to evaluate the impact of a multidisciplinary audit and feedback AMS intervention to improve future antibiotic prescribing<br>• The AMS included monthly feedback meetings at 13 PHCs using 10 prescriptions randomly selected for peer review<br>• Antibiotic prescriptions were subsequently scored for adherence to seven key measures including antibiotic choices vs. recommendations in STGs	• There was suboptimal adherence to the current STGs at the start of the study at only 11%<br>• Encouragingly, adherence increased to 53% over a 2-year period<br>• However, adherence to STGs was significantly lower in the winter and spring - concurrent with higher antibiotic prescribing/consumption. This potentially reflects inappropriate antibiotic prescribing for acute viral ARIs during these months<br>• Only 19% of prescriptions were correct in the first 6 months. This rose toa mean of 47% correct prescriptions in the last 6 months of the study ($p<0.001$) following active interventions.<br>• Overall, the AMS was associated with a 19.3% decrease in antibiotic consumption during the study period

Author and year	Aim and methods	Summary of key findings
	• Antibiotic utilisation patterns were also assessed	
Guma et al., 2022 [77]	• A semi-structured web-based questionnaire was used to assess current antibiotic empiric prescribing rates among 209 private GPs for their patients attending with ARIs and associated key factors	• 55.5% of surveyed GPs admitted to prescribing antibiotics empirically for patients with ARIs more than 70% of the time - primarily for symptom relief and the prevention of complications<br>• Encouragingly, GPs with more experience and working alone were slightly less likely to prescribe antibiotics empirically<br>• Key factors significantly associated with empiric prescribing of antibiotics were workload/time pressures, diagnostic uncertainty and the use of a formulary
Lagarde and Blaauw, 2023 [47]	• The objective was to assess prescribing practices for young and healthy SPs presenting to PHCs with viral bronchitis including both private (99 SPs) and public PHCs (102 SPs)<br>• Alongside this, 125 providers (across both sectors) were also interviewed face-to-face using a structured questionnaire	• Antibiotics were recommended in 72.6% of SP consultations, higher in the public sector (78.4%) vs. private sector (66.7%) - enhanced by perceived patient pressure<br>• These high rates were despite 84% of prescribers knowing the SP case was likely to be a viral infection (88% in the private sector vs. 77% in the public sector) and 58% knowing that antibiotics would not hasten recovery (40% public vs. 68% private)<br>• 47% of prescribers in public PHCs thought patients would not come back if they did not prescribe an antibiotic – higher in the private sector at 72% - despite SPs not demanding antibiotics<br>• Encouragingly, antibiotic prescribing rates were lower in both sectors (20% lower) in a previous study when HCPs were explicitly told by their patients that they did not want antibiotics for their infection unless they were necessary
Wieters et al., 2024 [48]	• The aim was to assess self-reported antibiotic use among 19 700 patients visiting healthcare facilities with acute RTI, acute gastrointestinal infection (GI) and acute febrile disease of unknown cause (AFDUC)<br>• The study was conducted in 4 African countries (Côte d'Ivoire, Burkina Faso, Democratic Republic of Congo, and South Africa)	• Out of the 7 258 (36.8%) patients who had taken antibiotics in the previous 10 days, 41.5% were prescribed for RTIs, 30.3% for AFDUC and 22.6% for GI infections. There were similar rates for RTIs in South Africa at 41.4% and AFDUC at 27.8%<br>• The most common antibiotic prescribed in the study was ceftriaxone (31.7% of antibiotics prescribed – lower in South Africa)<br>• Among patients with RTIs, the prescribing of ampicillin was highest in South Africa (22.8%) – with ceftriaxone at 15%
Chigome et al, 2025 [84]	• Multiple PPS were conducted among PHCs in two provinces in South Africa, as part of a larger global study	• Data for 615 patients were recorded in the PPS study with the most common symptoms for antibiotics being a genital discharge (21.8%), painful urination (18.4%), acute cough (17.7%), and a sore throat (13.5%), with patients potentially having more than one symptom

Author and year	Aim and methods	Summary of key findings
		• At least one antibiotic was prescribed for 87% of attending patients<br>• Access antibiotics accounted for 53.4% of antibiotics prescribed, with 46.6% being from the Watch group. Ceftriaxone (29.7%), amoxicillin (29.4%) and azithromycin (28.4%) were the most prescribed antibiotics<br>• Overall, there are considerable concerns with current prescribing practices among PHCs in South Africa
Maluleke et al., 2025 [108]	• A piloted questionnaire was administered to patients exiting 3 types of community pharmacies in a rural province<br>• Only patients dispensed a medicine (including over-the-counter medicines) were interviewed	• 465 patients were interviewed, with 54.4% dispensed at least one antibiotic<br>• 78.7% of dispensed antibiotics were without a prescription from either independent or franchise pharmacies<br>• STIs were the most common infectious disease for which an antibiotic was dispensed (60.1%) - 99.6% of these dispensed without a prescription<br>• URTIs were the most common infection where antibiotics were dispensed - 60.0% of the total, with little dispensing of antibiotics without a prescription (only 7.1%)
Van Hecke et al, 2024 [86]	• The objective was to assess the impact of a pharmacist-prescriber partnership to review antibiotic prescribing in public PHCs<br>• 457 patients with acute coughs were enrolled at 5 PHCs.	• 84% of patients enrolled in the review were prescribed an antibiotic for their acute cough<br>• The most prescribed antibiotics for these patients were amoxicillin (63%), co-amoxiclav (13%) and phenoxymethylpenicillin (6%), with a diagnosis of 'community-acquired pneumonia' the principal indication (35%)<br>• There was also a significant proportion of patients prescribed an antibiotic for 'acute cough' which needs addressing with future AMS activities including community pharmacists to reduce AMR
